# Supplementary material for: Multi-layered genetic approaches to identify approved drug targets
Source: Cell Genom. 2023 Jun 15;3(7):100341. doi: 10.1016/j.xgen.2023.100341 (PMC10363916; doi:10.1016/j.xgen.2023.100341)
Supplement: Document S1. Figures S1–S8 and Tables S14 and S15 [file mmc1.pdf]

Cell Genomics, Volume *3*

Supplemental information

Multi-layered genetic approaches

to identify approved drug targets

Marie C. Sadler, Chiara Auwerx, Patrick Deelen, and Zoltán Kutalik

Supplemental information

Multi-layered genetic approaches to identify approved drug targets

Marie C. Sadler, Chiara Auwerx, Patrick Deelen, Zoltán Kutalik

Supplemental Figures

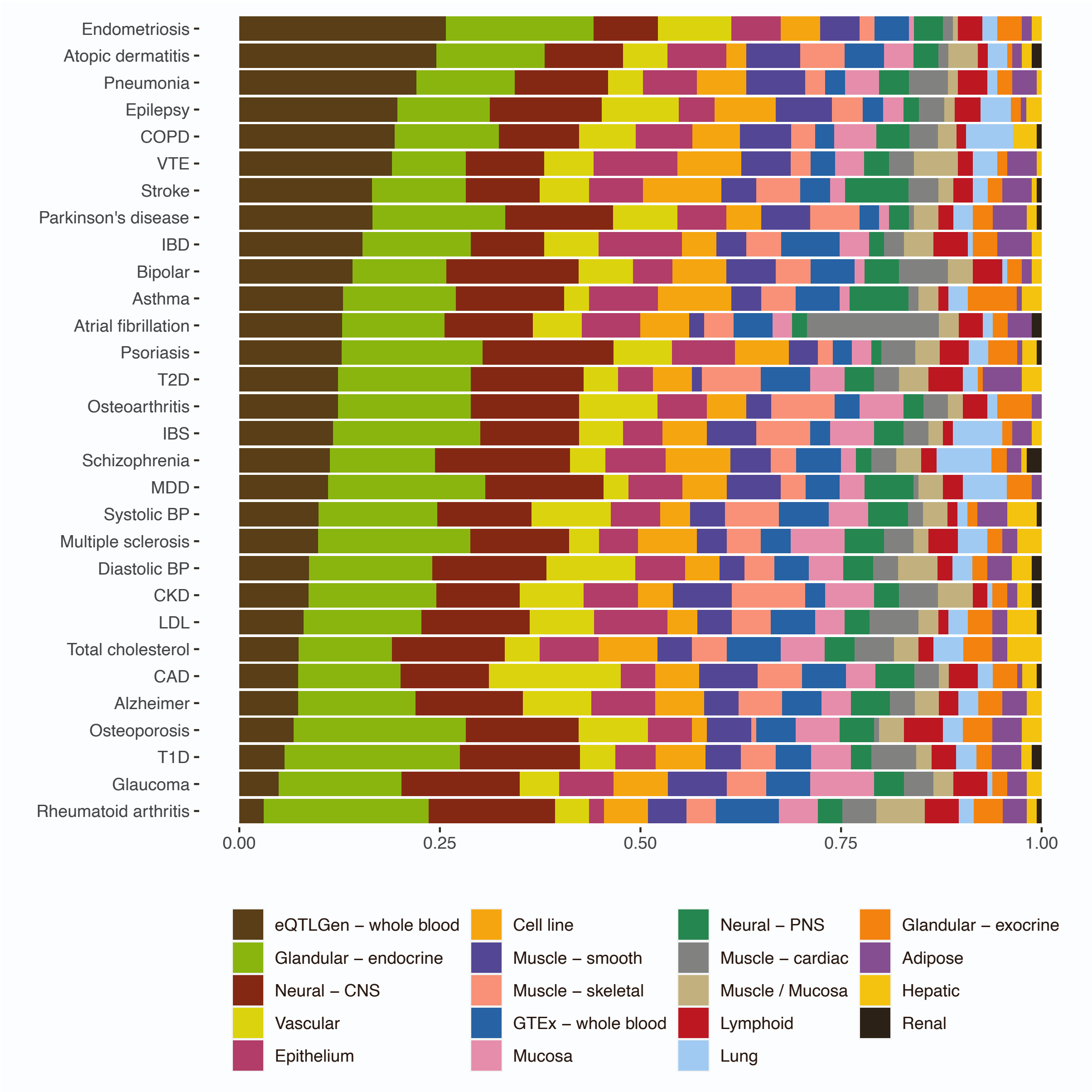

Figure S1. Gene-tissue mapping proportions.
Proportion of genes mapped to a particular tissue category in the tissue-wide expression quantitative trait locus (eQTL)-genome-wide association analysis (GWAS) analysis. For each gene, the tissue with the lowest Mendelian randomization (MR) p-value was selected. Tissue category belonging are shown in Table S4 and numerical proportion values in Table S5. This figure is related to the eQTL-GWAS method in Figures 2,3 and 5.

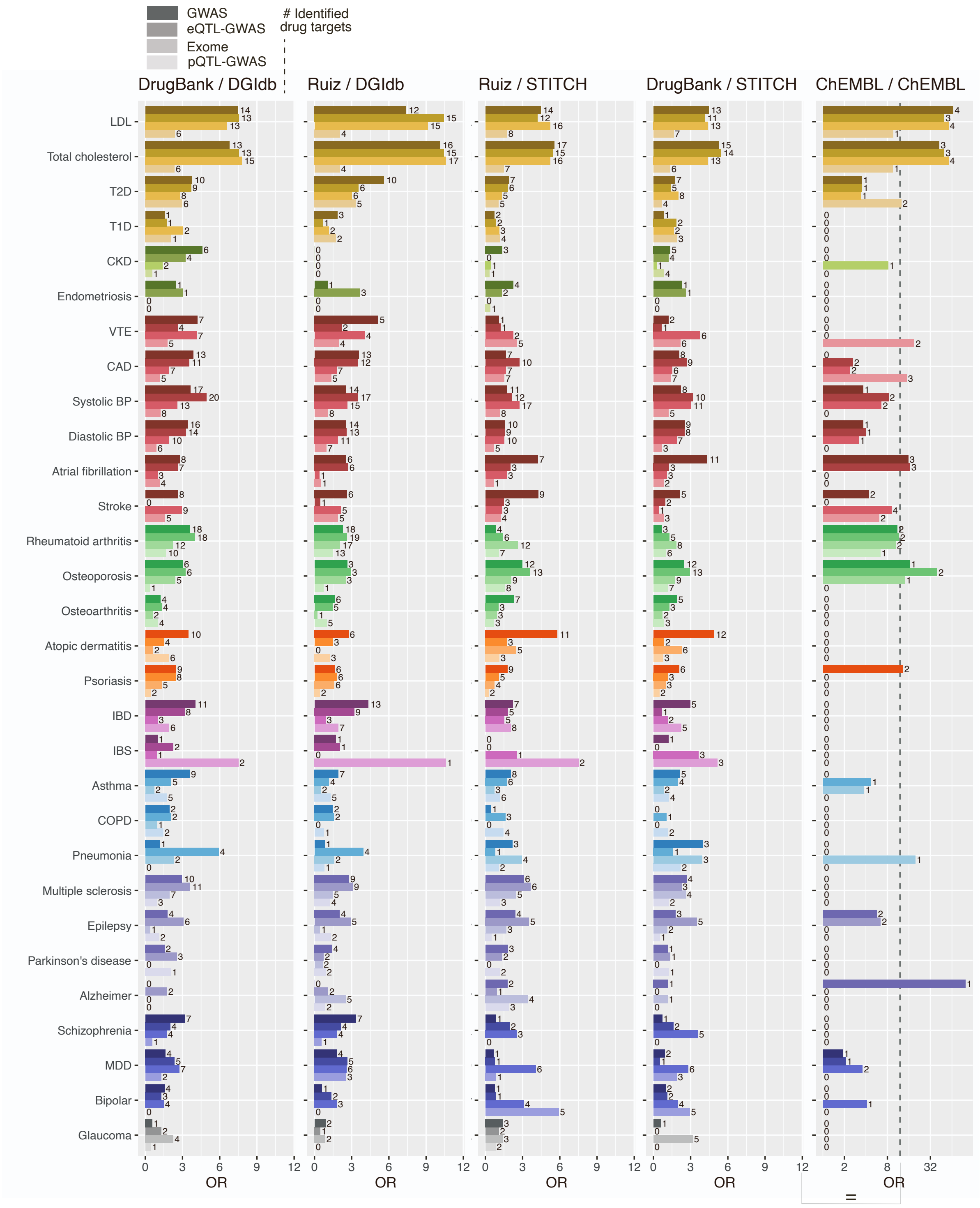

Figure S2. **Enrichment for drug target genes across drug databases.**

Barplots with odds ratios (ORs) calculated from Fisher's exact tests between drug target genes and prioritized genes for the four tested methods and thirty traits. Prioritized genes were defined as the top 1% percentile of the GWAS, eQTL-GWAS and Exome methods, and 5% of the pQTL-GWAS method. Drug target genes were defined by the drug database combinations (drug-indication and drug-target links) shown in the title of each barplot. Only drug target genes that could be tested by the respective method were considered. The number on the right of each bar indicates the number of identified drug target genes. In the barplot corresponding to the ChEMBL/ChEMBL database, the x-axis is log-transformed and therefore ORs of 0 (i.e., no identified drug target) were set to 1. This figure is related to Figure 3A which shows enrichment for the DrugBank /DGIdb combination.

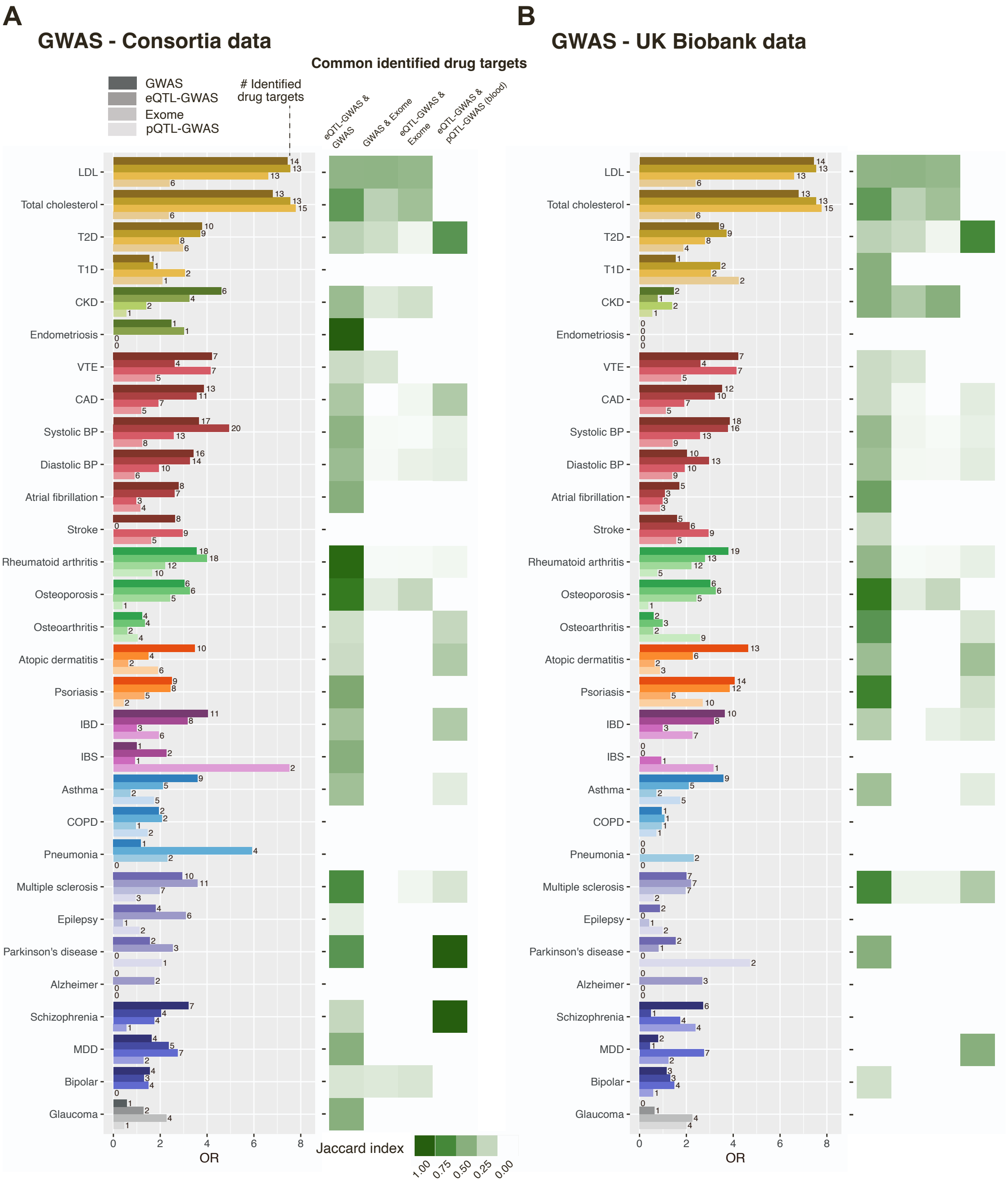

Figure S3. **Comparing consortia and UK Biobank GWAS data in drug target enrichment analyses.**
(A) Enrichment analysis using consortia GWAS summary statistics in the GWAS, eQTL-GWAS and pQTL-GWAS methods.
(B) Enrichment analysis using UKBB GWAS summary statistics in the GWAS, eQTL-GWAS and pQTL-GWAS methods. The Exome analysis is only performed on UK Biobank data. Left: Barplot with odds ratios (ORs) calculated from Fisher's exact tests between drug target genes and prioritized genes for the four tested methods and thirty traits. Prioritized genes were defined as the top 1% percentile of the GWAS, eQTL-GWAS and Exome methods, and 5% of the pQTL-GWAS method. Drug target genes were defined from the DrugBank and DGIdb databases, and only drug target genes that could be tested by the respective method were considered. The number on the right of each bar indicates the number of identified drug target genes. Right: Overlap of identified drug target genes between pairs of methods quantified through the Jaccard index. The blood-only eQTL-GWAS gene prioritization

method was used for the comparison with the pQTL-GWAS method. This figure is related to Figure 3A which shows enrichment for drug targets using consortia GWAS.

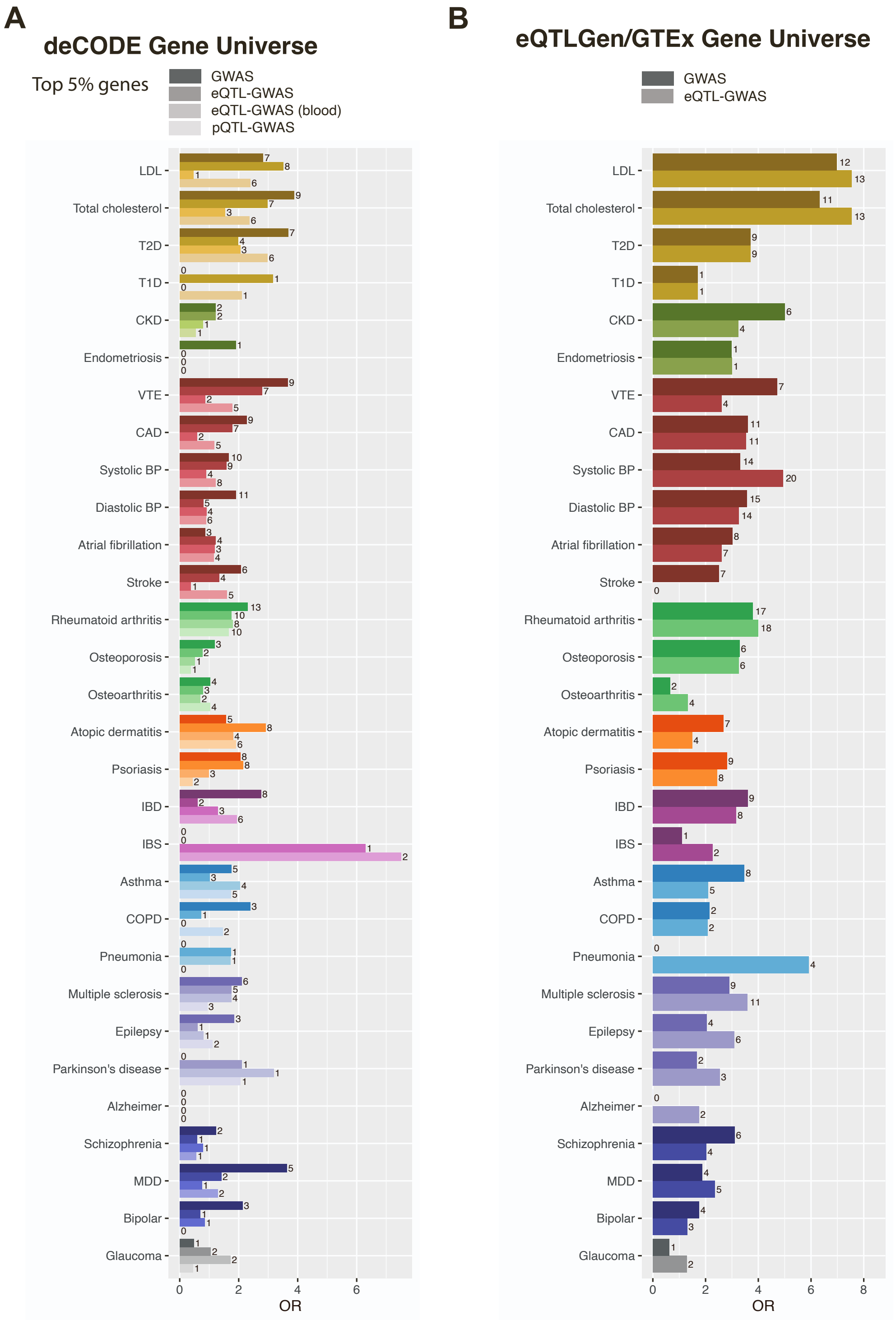

Figure S4. **Enrichment for drug target genes using the same background genes.**

(A) Enrichment analysis was performed by subsetting the gene universe of the GWAS and eQTL-GWAS (tissue-wide and whole blood only) methods to the genes available in the deCODE study (i.e., proteins used in the pQTL-GWAS analysis).

(B) Enrichment analysis was performed by subsetting the gene universe of the GWAS method to the genes available in the tissue-wide eQTL-GWAS analysis. Both plots show barplots with odds ratios (ORs) calculated from Fisher's exact tests between drug target genes and prioritized genes for the four tested methods and thirty traits. Drug target genes were defined from the DrugBank and DGIdb databases, and only drug target genes that could be tested by the respective method were considered.

The number on the right of each bar indicates the number of identified drug target genes. This figure is related to Figure 3A in which background genes were different among methods.

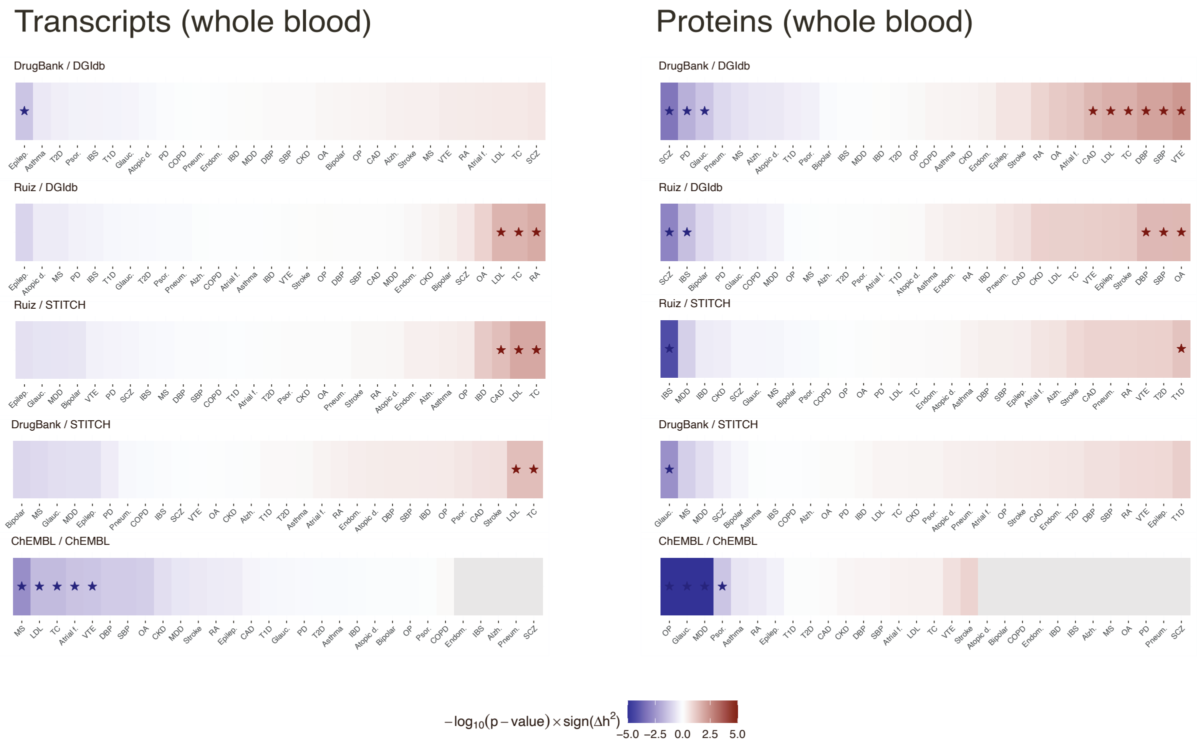

Figure S5. **Heritability of drug target genes.**

Difference in *cis*-heritability of drug target compared to non-drug target measured transcript and protein levels. For each trait, the difference in heritability was calculated through a two-sided t-test. When the difference was negative (i.e., drug target genes were less heritable), the -$\log_{10}$(p-value) is plotted in blue, otherwise in red. Traits for which the difference was nominally significant (p-value < 0.05), are indicated with a star. If less than three drug target genes could be tested for a trait, a grey box is plotted. This figure is related to the result section “Heritability of drug target transcripts and proteins”.

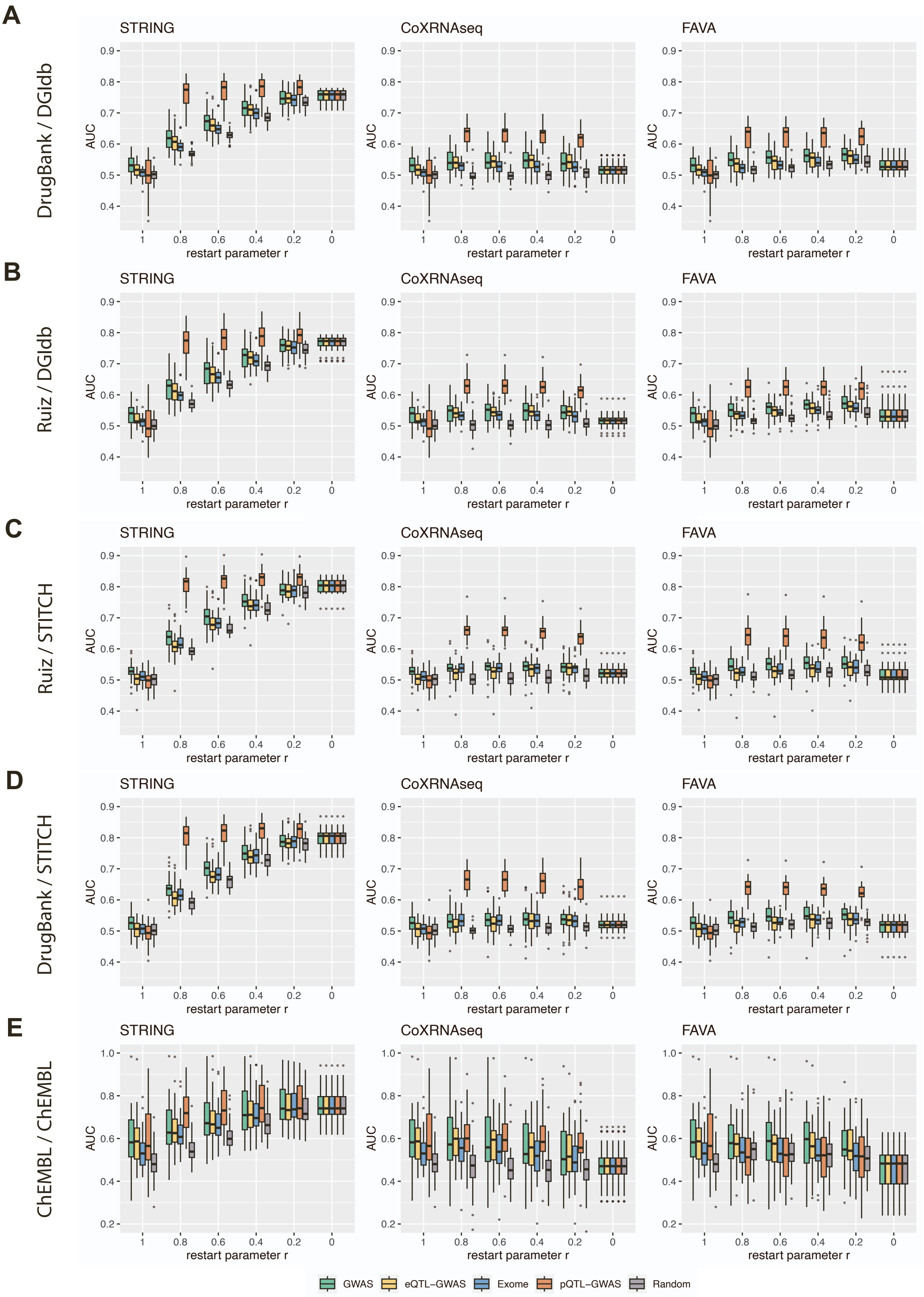

Figure S6. Effect of network diffusion to prioritize drug target genes across drug databases (AUC values).

Boxplots showing the area under the receiver operating characteristic curve (AUC) values for each network type (STRING, CoXRNAseq and FAVA) and method at different restart parameter values *r*.

AUC values were calculated for each of the thirty traits, and drug target genes were defined by the respective drug database combination (drug-indication and drug-target links, (A)-(E). The boxplots bound the 25th, 50th (median, centre), and the 75th quantile. Whiskers range from minima (Q1 – 1.5 • IQR) to maxima (Q3 + 1.5 • IQR) with points above or below representing potential outliers. This figure is related to Figure 5A which shows AUC values for the DrugBank /DGIdb combination.

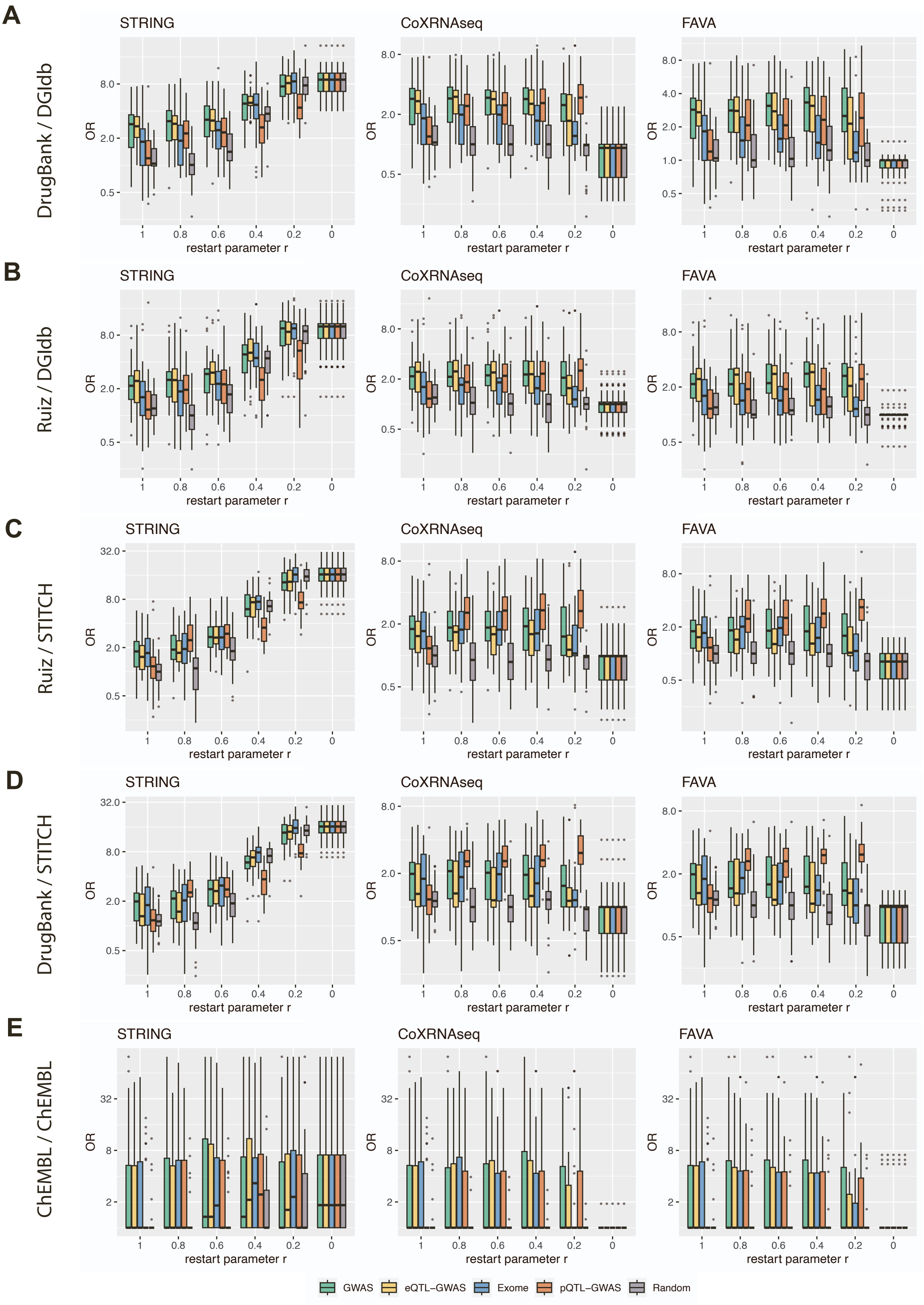

Figure S7. Effect of network diffusion to prioritize drug target genes across drug databases (ORs). Odds ratios (ORs) between prioritized genes (top 1%) and drug target genes for each network type (STRING, CoXRNAseq and FAVA) and method at different restart parameter values *r*. Drug target genes

were defined by the respective drug database combination (drug-indication and drug-target links, (A)-(E)). The OR was set to 1 for traits with no identified drug target genes. The boxplots bound the 25th, 50th (median, centre), and the 75th quantile. Whiskers range from minima (Q1 – 1.5 • IQR) to maxima (Q3 + 1.5 • IQR) with points above or below representing potential outliers. This figure is related to Figure 5B which shows ORs for the DrugBank /DGIdb combination.

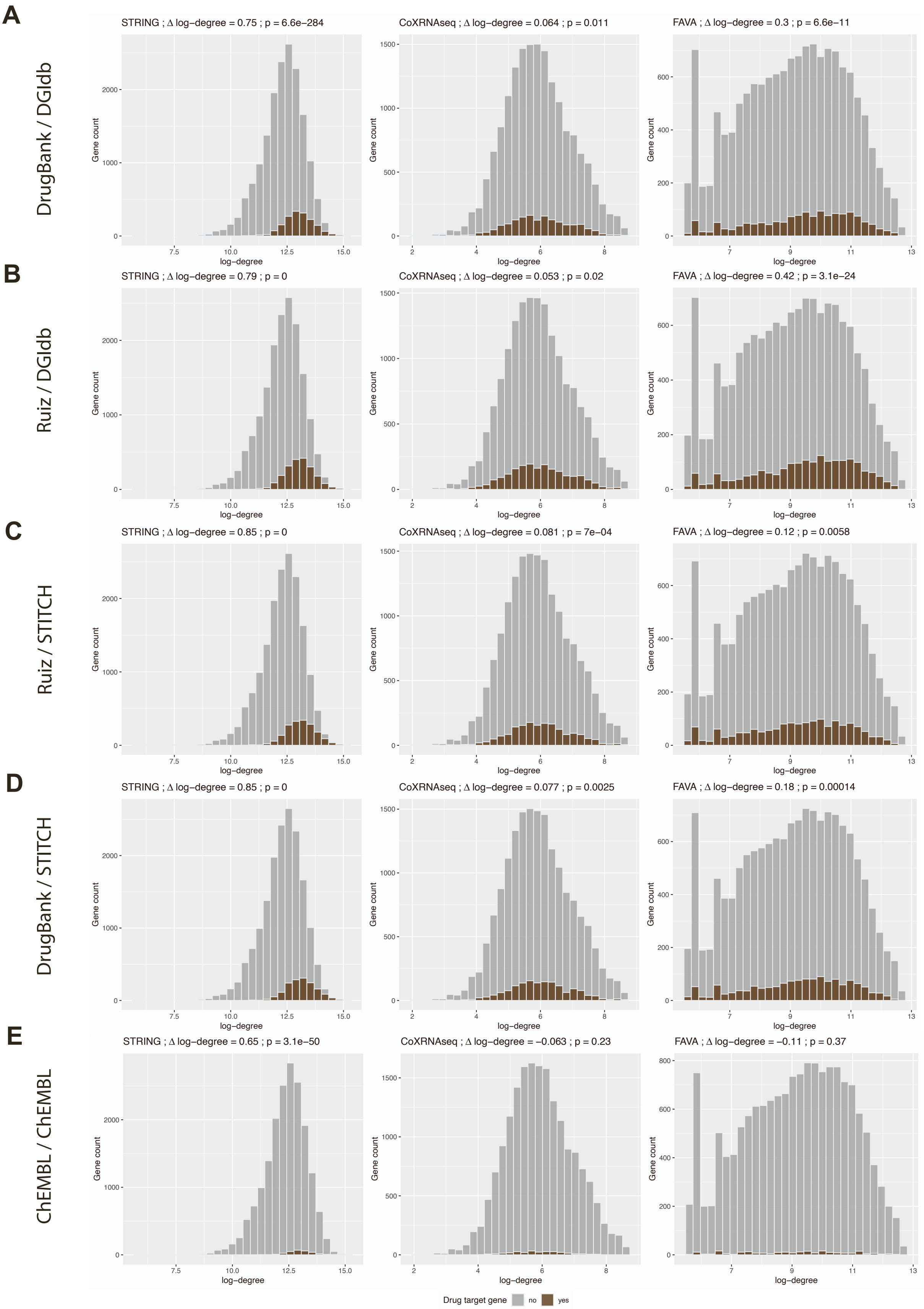

Figure S8. **Network degree distribution of drug target genes.**

Histograms showing the degree distribution of drug target genes and non-drug target genes in each network across drug databases (drug-indication and drug-target links, (A)-(E)). The difference in log-

degree and the p-values from two-sided t-tests are shown in the title. This figure is related to Figure 5C which shows network degree distributions for the DrugBank /DGIdb combination.

Supplemental Tables

Table S14 . Network properties.

Network properties of the weighted networks that were analysed in this study (STRING: protein-protein interaction network, FAVA: co-expression network including proteomics, CoXRNAseq: co-expression network); related to STAR Methods section "Networks".

Nodes: number of nodes (genes) in the network.
Edges: number of total edges in the network.
Median degree: median degree in the network (i.e., weighted node degree)
Average log-degree: mean log-degree in the network
sd log-degree: standard deviation of the log-degree in the network

Network	STRING	FAVA	CoXRNAseq
**Nodes**	18573	15829	18695
**Edges**	11136598	951878	1119670
**Median degree**	257968.00	10672.79	356.44
**Average log-degree**	12.35	9.12	5.93
**sd log-degree**	0.96	1.73	1.02

Supplementary Table 15: Enrichment of testable and measured proteins for drug target genes.

Two-sided binomial test results to determine the enrichment of testable (~1,870, proteins that had at least 1 pQTL) and measured (~4,450) proteins for drug target genes among all protein-coding genes; related to STAR Methods section "Enrichment of proteins for drug targets".

Drug database	Observed proportion	Expected proportion	Pval	Set
Ruiz/DGIdb	0.2057	0.0964	2.05E-49	Testable
Ruiz/STITCH	0.2172	0.0964	2.49E-51	Testable
DrugBank/DGIdb	0.2164	0.0964	1.30E-47	Testable
DrugBank/STITCH	0.2246	0.0964	3.20E-50	Testable
ChEMBL/ChEMBL	0.1324	0.0964	5.04E-02	Testable
Ruiz/DGIdb	0.4045	0.2291	1.80E-69	Measured
Ruiz/STITCH	0.4689	0.2291	3.88E-108	Measured
DrugBank/DGIdb	0.4171	0.2291	1.15E-64	Measured
DrugBank/STITCH	0.4820	0.2291	3.76E-105	Measured
ChEMBL/ChEMBL	0.3015	0.2291	5.98E-03	Measured
